# The metabolic and renal effects of adrenaline and milrinone in patients with myocardial dysfunction after coronary artery bypass grafting

**DOI:** 10.1186/cc5904

**Published:** 2007-04-30

**Authors:** Matthias Heringlake, Marit Wernerus, Julia Grünefeld, Stephan Klaus, Hermann Heinze, Matthias Bechtel, Ludger Bahlmann, Jochen Poeling, Julika Schön

**Affiliations:** 1 Department of Anesthesiology, Universität zu Lübeck, D-23538 Lübeck, Germany; 2 Department of Anesthesiology, Herz-Jesu Krankenhaus Münster-Hiltrup, D – 48165 Münster, Germany; 3 Department of Cardiac Surgery, Universität zu Lübeck, D-23538 Lübeck, Germany; 4 Department of Anesthesiology, Krankenhaus Weser-Egge, D – 37671 Höxter, Germany; 5 Department of Cardiac Surgery, Schüchtermann-Klinik, D – 49214 Bad Rothenfelde, Germany

## Abstract

**Introduction:**

Myocardial dysfunction necessitating inotropic support is a typical complication after on-pump cardiac surgery. This prospective, randomized pilot study analyzes the metabolic and renal effects of the inotropes adrenaline and milrinone in patients needing inotropic support after coronary artery bypass grafting (CABG).

**Methods:**

During an 18-month period, 251 patients were screened for low cardiac output upon intensive care unit (ICU) admission after elective, isolated CABG surgery. Patients presenting with a cardiac index (CI) of less than 2.2 liters/minute per square meter upon ICU admission – despite adequate mean arterial (titrated with noradrenaline or sodium nitroprusside) and filling pressures – were randomly assigned to 14-hour treatment with adrenaline (*n *= 7) or milrinone (*n *= 11) to achieve a CI of greater than 3.0 liters/minute per square meter. Twenty patients not needing inotropes served as controls. Hemodynamics, plasma lactate, pyruvate, glucose, acid-base status, insulin requirements, the urinary excretion of alpha-1-microglobuline, and creatinine clearance were determined during the treatment period, and cystatin-C levels were determined up to 48 hours after surgery (follow-up period).

**Results:**

After two to four hours after ICU admission, the target CI was achieved in both intervention groups and maintained during the observation period. Plasma lactate, pyruvate, the lactate/pyruvate ratio, plasma glucose, and insulin doses were higher (*p *< 0.05) in the adrenaline-treated patients than during milrinone or control conditions. The urinary excretion of alpha-1-microglobuline was higher in the adrenaline than in the control group 6 to 14 hours after admission (*p *< 0.05). No between-group differences were observed in creatinine clearance, whereas plasma cystatin-C levels were significantly higher in the adrenaline than in the milrinone or the control group after 48 hours (*p *< 0.05).

**Conclusion:**

This suggests that the use of adrenaline for the treatment of postoperative myocardial dysfunction – in contrast to treatment with the PDE-III inhibitor milrinone – is associated with unwarranted metabolic and renal effects.

**Clinical trials registration:** ClinicalTrials.gov NCT00446017.

## Introduction

Postoperative myocardial dysfunction necessitating inotropic support is a typical complication after on-pump cardiac surgery [1]. The ideal pharmacological treatment of a postoperative low cardiac output state is a matter of ongoing debate [2] and differs markedly among European countries. Whereas a study by Leone and coworkers [3] reported that dobutamine is the preferred agent for the treatment of myocardial dysfunction in France, a recent survey performed in Germany revealed that the first-line inotrope in German heart centers is adrenaline in 41.8% of cases, followed by dobutamine in 30.9%, and phosphodiesterase III (PDE-III) inhibitors like milrinone and enoximone in 14.9% [4].

Observational data in critically ill patients with septic conditions suggest that the use of adrenaline is associated with a worse outcome [5]. This may be explained, at least in part, by the effects of this drug on glucose and lactate homeostasis [6], leading to hyperglycemia and hyperlactacidemia, and its effects on regional blood flow, leading to a decrease in splanchnic perfusion [7]. In line with this, we have recently shown that the preference for the use of adrenaline in vasopressor doses is associated with a higher incidence of renal dysfunction in patients undergoing cardiac surgery [8].

No adverse metabolic effects have been reported during treatment with PDE-III inhibitors. Moreover, preemptive therapy with PDE-III inhibitors has been shown to exert beneficial effects on markers of renal tubular injury in patients undergoing on-pump cardiac surgery [9,10]. In contrast, data derived from patients with decompensated heart failure suggest that the use of milrinone may be associated with a higher rate of renal dysfunction [11].

The present prospective, randomized pilot study thus explores the metabolic and renal effects of treating a postoperative low output state with either adrenaline or milrinone in comparison with a group of patients not needing inotropic support after on-pump coronary artery bypass grafting (CABG) surgery.

## Materials and methods

Following approval by the local ethical committee and preoperative written consent, 251 consecutive patients scheduled for elective CABG were screened for postoperative low cardiac output syndrome (LCOS) during an 18-month period. LCOS was defined as a cardiac index (CI) of less than 2.2 liters/minute per square meter despite the fact that filling pressures had been optimized by colloid infusion with gelatine polysuccinate to a central venous pressure (CVP) of 12 to 15 mm Hg and a diastolic pulmonary artery pressure of 15 to 18 mm Hg *and *that mean arterial blood pressure (MAP) had been titrated to 65 to 90 mm Hg by administration of noradrenaline or sodium nitroprusside.

Sixty-eight patients needed intraoperative inotropic support, and 107 fulfilled the control criteria (CI of greater than 3.0 liters/minute per square meter). Intraoperative protocol violations (mostly by withholding the pulmonary artery catheter) occurred in 36 patients. Postoperative LCOS was observed in 40 patients, of whom 22 were not randomly assigned by the intensivist in charge, leaving 18 patients to be randomly treated postoperatively according to the study protocol.

Upon fulfilling the inclusion criteria, patients were treated open-label with either adrenaline (ADR: *n *= 7) or milrinone (MIL: *n *= 11) to achieve and maintain a target CI of greater than 3.0 liters/minute per square meter throughout the treatment period. Drugs were applied by continuous infusion without a bolus. Twenty patients not needing inotropes served as controls (CON). Randomization was performed by the sealed envelope technique.

Treatment in the intensive care unit (ICU) and in the intermediate care unit was at the discretion of the physicians and nurses in charge. With the exception of the hemodynamic goals given above and the prohibition of using diuretics or hydroxyethylstarch preparations during the treatment period, no specific therapeutic orders were given. Standard postoperative care included targeting of blood glucose levels of less than 200 mg/dl by continuous infusions of insulin and targeting of hemoglobin levels of greater than 80 mg/l by infusion of packed red cells.

During a period of 15 months, it became clear that the predefined number of 20 patients per group could not be accomplished within the predefined period of 18 months. Thus, during the last three months of the study period, patients fulfilling the inclusion criteria were also randomly assigned *intraoperatively *after weaning from cardiopulmonary bypass (CPB) (ADR: *n *= 5; MIL: *n *= 6). These patients are not included in the present analysis. The study was terminated after 18 months because it became clear that the enrollment of a larger series of patients could not be accomplished within an acceptable time frame.

### Surgical procedures and monitoring

Routine CPB grafting was performed in moderate hypothermia using antegrade blood cardioplegia. All patients were equipped with a radial arterial line, a central venous catheter, and a pulmonary artery catheter for continuous determination of mixed venous oxygen saturation (SvO_2_) and CI (Vigilance; Edwards Lifesciences LLC, Irvine CA, USA). All patients received a bolus of 500 mg of methylprednisolone before CPB and 4 MU of aprotinin throughout the surgical procedure.

### Measurements

Hemodynamics and renal function were studied during the 14-hour treatment period after admission to the ICU. Hemodynamics were recorded every two hours. Hemodynamic variables determined included MAP, heart rate (HR), CVP, systolic pulmonary artery pressure, mean pulmonary artery pressure, and diastolic pulmonary artery pressure, SvO_2_, and CI.

Urine was sampled at baseline (t0) and at 2, 6, 10, and 14 hours. Measured variables included urine flow (UV), urinary excretion of sodium, urinary excretion of creatinine, and urinary excretion of alpha-1-microglobuline (A1-MG_U_).

Arterial blood samples for determination of lactate, pyruvate, glucose, and creatinine were taken after ICU admission (t0) and at 2, 6, 10, 14, and 48 hours thereafter. Plasma concentrations of cystatin-C (Cys-C) were determined at t0, t14, and t48.

### Calculations

The lactate/pyruvate ratio (LPR), the fractional excretion of sodium, and creatinine clearance (C_Crea_) were calculated according to standard formula. A1-MG_U _is given relative to the urinary creatinine concentration.

### Analytical methods

Lactate, pyruvate, and glucose were determined enzymatically on a CMA-600 analyzer (CMA Microdialysis AB, Solna, Sweden) as described in detail elsewhere [12]. The detection limit for lactate was 0.1 mmol/l, for pyruvate 10 μmol/l, and for glucose 0.1 mmol/l (1.8 mg/dl).

Cys-C was determined by an enzyme-linked immunosorbent assay for human Cys-C (United States Biological Inc., Swampscott, MA, USA). The sensitivity was 69.2 ng/ml. The intra-assay coefficients of variation (*n *= 8) were 9.6% and 5% at 589 and 2,863 ng/ml, respectively. The inter-assay coefficients of variation (*n *= 8) were 5.2% and 4.8% at 600 and 2,905 ng/ml, respectively. The recovery rate was between 81.9% and 90%.

A1-MG was determined nephelometrically using an antiserum against human A1-MG (Dade Behring Marburg GmbH, Marburg, Germany). The intra-assay coefficients of variation (*n *= 21) are 5.2% and 2.9% for concentrations of 7.7 and 41.8 mg/l, respectively. The inter-assay coefficients of variation are 13.2% and 7.4% for concentrations of 6.8 and 42 mg/l, respectively.

### Statistical analysis

If not stated otherwise, data are given as mean ± standard deviation. Following analysis by Kolmogorov-Smirnov test for normality of distribution, differences in comparison with baseline were analyzed by Student paired *t *test. Between-group differences were determined by analysis of variance with *post hoc *Fisher's predicted least-square difference (continuous data) or chi-square test (nominal variables), as appropriate. A *p *value of less than 0.05 was considered to indicate significance.

## Results

Demographic data, preoperative left ventricular ejection fraction, and procedure-related data were comparable between the groups (Table 1). The time course of hemodynamics is given in Table 2, showing that CI was effectively increased in both intervention groups within two to four hours. The ADR group received 1.2 ± 0.5 mg of adrenaline and 0.6 ± 1.6 mg of noradrenaline, and the MIL group received 21.6 ± 7.7 mg of milrinone and 0.7 ± 0.8 mg of noradrenaline. The noradrenaline use in the CON group was 0.2 ± 0.4 mg and thereby lower than in the MIL group (*p *< 0.05). No between-group differences were observed in the amount of crystalloid (ADR: 1,098 ± 288 ml; MIL: 1,182 ± 262 ml; CON: 1,054 ± 266 ml) and colloidal (ADR: 1,928 ± 607 ml; MIL: 1,937 ± 562 ml; CON: 1,625 ± 666 ml) fluids during the observation period of 14 hours. Transfusion requirements for packed red cells were not different between the groups (ADR: 250 ± 322 ml; MIL: 204 ± 245 ml; CON: 87 ± 167 ml), whereas requirements for fresh frozen plasma showed a trend (*p *= 0.06) for a higher use in the ADR and MIL groups (ADR: 107 ± 196 ml; MIL: 68 ± 161 ml; CON: 0 ± 0 ml).

**Table 1:** Demographic and procedure-related variables

	Adrenaline *n *= 7	Milrinone *n *= 11	Control *n *= 20
Age (years)	65 ± 9	69 ± 9	63 ± 9
Height (centimeters)	169 ± 7	174 ± 7	176 ± 7
Weight (kilograms)	79 ± 15	90 ± 15	85 ± 13
Ejection fraction (percentage)	64 ± 16	52 ± 19	61 ± 16
Creatinine (micromoles per liter)	97 ± 22	97 ± 30	84 ± 17
Diabetics	*n *= 3/7	*n *= 3/11	*n *= 4/20
Operation time (minutes)	228 ± 33	215 ± 31	211 ± 41
Cardiopulmonary bypass (minutes)	89 ± 19	89 ± 25	80 ± 19
Aortic crossclamping (minutes)	64 ± 16	59 ± 17	59 ± 17

Demographic and surgical data of patients presenting with myocardial dysfunction (cardiac index of less than 2.2 liters/minute per square meter despite optimization of mean arterial pressure and filling pressures) upon intensive care unit admission and of control patients not needing inotropic support after coronary artery bypass grafting with cardiopulmonary bypass. Analysis of variance with Fisher's predicted least-square difference (continuous variables) and chi-square test (categorical variables) revealed no significant between-group differences.

**Table 2:** Time course of hemodynamic variables and plasma bicarbonate levels

		t0	t2	t4	t6	t8	t10	t12	t14
CI (liters per minute per square meter)	ADR	1.9 ± 0.2^b^	3.3 ± 0.6^a^	3.2 ± 0.4^a^	3.3 ± 0.6^a^	3.2 ± 0.5^a^	3.1 ± 0.5^a^	3.3 ± 0.6^a^	3.5 ± 0.5^a^
	MIL	2.0 ± 0.2^b^	2.6 ± 0.2^a^	3.0 ± 0.6^a^	2.9 ± 0.4^a^	3.1 ± 0.5^a^	3.1 ± 0.5^a^	3.5 ± 0.6^a^	3.3 ± 0.5^a^
	CON	3.3 ± 0.4	3.1 ± 0.4	3.3 ± 0.6	3.4 ± 0.6^a^	3.5 ± 0.8	3.5 ± 0.8^a^	3.8 ± 0.8^a^	3.8 ± 1.0^a^

SvO_2 _(percentage)	ADR	63.6 ± 5.7^b^	74.1 ± 7.6^a^	68.6 ± 6.2	69.9 ± 6.6	69.1 ± 6.7^a^	69.1 ± 6.7	68.0 ± 4.9	67.2 ± 6.2
	MIL	65.3 ± 8.5^b^	68.7 ± 5.4	68.6 ± 9.9	65.4 ± 5.9^b^	65.4 ± 5.9	68.8 ± 7.7^a^	66.1 ± 6.6	65.7 ± 6.2
	CON	74.1 ± 5.5	73.5 ± 5.0	71.9 ± 4.5	71.9 ± 4.5	71.9 ± 4.5	71.2 ± 6.6	71.3 ± 5.3	71.5 ± 5.2

MAP (millimeters of mercury)	ADR	79 ± 8	70 ± 7^abc^	73 ± 9^b^	76 ± 6	78 ± 7	80 ± 13	82 ± 10	82 ± 10
	MIL	80 ± 13	83 ± 12	79 ± 11	79 ± 9	81 ± 7	79 ± 9	82 ± 10	77 ± 8
	CON	83 ± 8	80 ± 8	86 ± 10	82 ± 11	82 ± 8	82 ± 8.0	80 ± 8	79 ± 9

HR (beats per minute)	ADR	97 ± 11	97 ± 8	97 ± 6	95 ± 10	95 ± 10	96 ± 10	96 ± 11	99 ± 10
	MIL	92 ± 10	91 ± 12	90 ± 11	101 ± 14^a^	101 ± 14^a^	100 ± 11^a^	102 ± 10^a^	102 ± 9^a^
	CON	95 ± 8	96 ± 5	96 ± 5	98 ± 6	97 ± 6	98 ± 7	99 ± 7^a^	98 ± 6^a^

PAP mean (millimeters of mercury)	ADR	24 ± 6	26 ± 4	24 ± 4	24 ± 6	24 ± 6	23 ± 6	23 ± 6	20 ± 6
	MIL	27 ± 5^b^	26 ± 5	26 ± 6	26 ± 6.4	24 ± 5	25 ± 5	25 ± 4	23 ± 3
	CON	23 ± 4	25 ± 5	25 ± 5^a^	23 ± 5	23 ± 5	21 ± 6	20 ± 7	21 ± 8

CVP (millimeters of mercury)	ADR	14 ± 4	14 ± 4	13 ± 3	13 ± 4	13 ± 4	12 ± 5	12 ± 5	10 ± 4
	MIL	16 ± 4^b^	14 ± 3^a^	14 ± 3	13 ± 4^a^	12 ± 4^a^	13 ± 5^a^	13 ± 4^a^	12 ± 4^a^
	CON	12 ± 4	12 ± 4	12 ± 4	11 ± 4	14 ± 3	10 ± 4	10 ± 3	10 ± 4

HCO_3_- (millimoles per liter)	ADR	22.6 ± 3.1	20.6 ± 3.7^a^	20.6 ± 3.6^a^	21.3 ± 2.9	22.6 ± 2.6	23.3 ± 2.2	23.7 ± 1.9	24.0 ± 1.7^bc^
	MIL	22.8 ± 2.4	22.4 ± 2.7	22.7 ± 4.4	22.1 ± 1.9	21.8 ± 2.0^a^	22.4 ± 2.4	22.3 ± 2.3	22.4 ± 1.9
	CON	22.5 ± 1.1	22.3 ± 1.1	22.0 ± 1.0^a^	22.1 ± 1.3	22.0 ± 1.2	22.3 ± 1.2	22.6 ± 1.3	22.5 ± 1.6

The time course of hemodynamics and plasma bicarbonate levels (HCO_3_-) in patients who presented with myocardial dysfunction (cardiac index [CI] of less than 2.2 liters/minute per square meter despite optimization of mean arterial pressure and filling pressures) upon ICU admission and who were treated with adrenaline (ADR) or milrinone (MIL) and of control patients (CON) not needing inotropic after coronary artery bypass grafting with cardiopulmonary bypass. ^a^Significant difference (*p *< 0.05) in comparison with baseline (t0); ^b^significant difference (*p *< 0.05) in comparison with the control group; ^c^significant difference (*p *< 0.05) between the adrenaline and the milrinone group. CVP, central venous pressure; HR, heart rate; MAP, mean arterial blood pressure; PAP, pulmonary artery pressure; SvO_2_, mixed venous oxygen saturation.

### Metabolism

**Figure 1:**
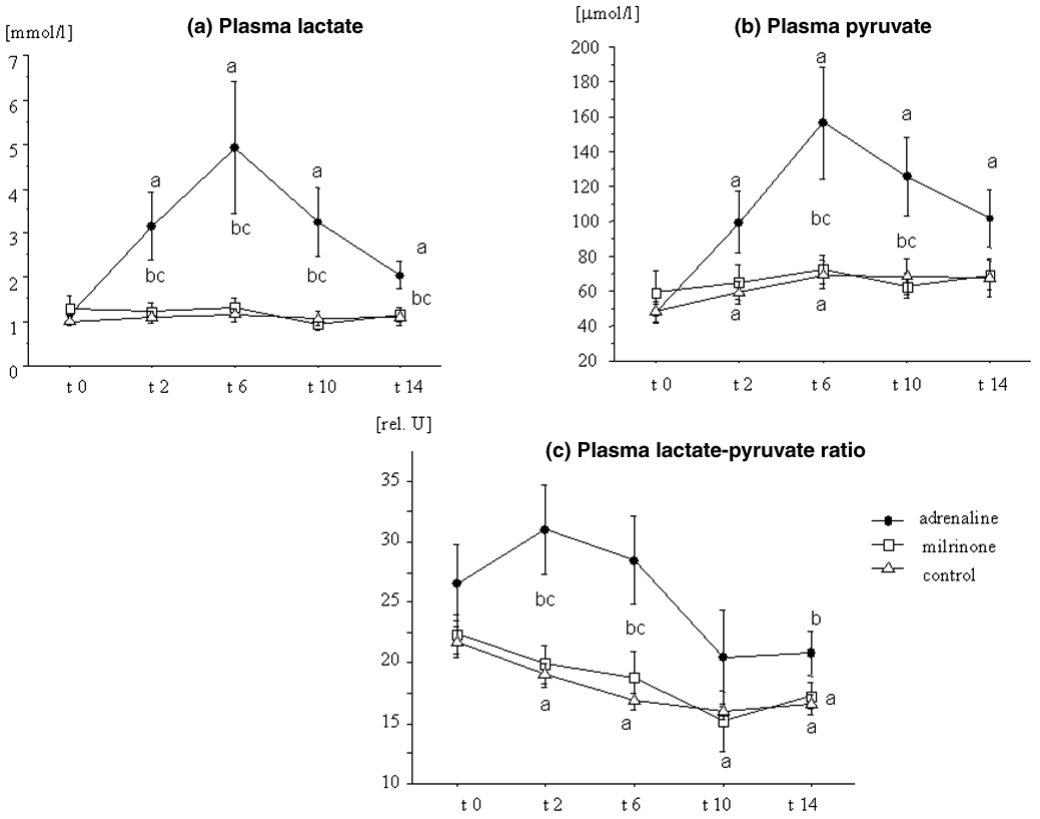
Lactate-pyruvate metabolism. The time course of plasma lactate **(a)**, pyruvate **(b)**, and the lactate/pyruvate ratio **(c) **in patients with myocardial dysfunction after coronary artery bypass grafting surgery, treated with adrenaline (*n *= 7) or milrinone (*n *= 11), and in control patients (*n *= 20) not needing inotropic support. Data are given as mean ± standard error of the mean. ^a^Significant difference (*p *< 0.05) in comparison with baseline values (paired *t *test); ^b^significant difference (*p *< 0.05) in comparison with the control group (analysis of variance [ANOVA] with *post hoc *Fisher's predicted least-square difference [PLSD]); ^c^significant difference (*p *< 0.05) between the adrenaline and the milrinone group (ANOVA with *post hoc *Fisher's PLSD).

**Figure 2:**
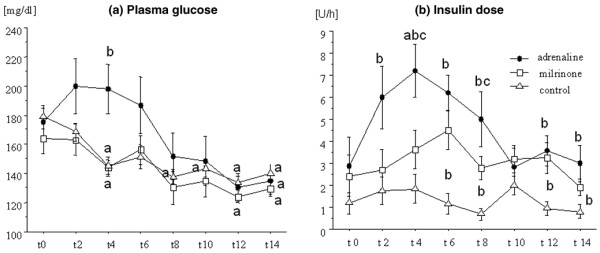
Plasma glucose and insulin doses. The time course of plasma glucose **(a) **and insulin **(b) **doses in patients with myocardial dysfunction after coronary artery bypass grafting surgery, treated with adrenaline (*n *= 7) or milrinone (*n *= 11), and in control patients (*n *= 20) not needing inotropic support. Data are given as mean ± standard error of the mean. ^a^Significant difference (*p *< 0.05) in comparison with baseline values (paired *t *test); ^b^significant difference (*p *< 0.05) in comparison with the control group (analysis of variance [ANOVA] with *post hoc *Fisher's predicted least-square difference [PLSD]); ^c^significant difference (*p *< 0.05) between the adrenaline and the milrinone group (ANOVA with *post hoc *Fisher's PLSD).

The time courses of plasma lactate, pyruvate, LPR, and glucose are given in Figures 1 and 2, showing that these parameters were significantly higher during the treatment period in the ADR than in the MIL or the CON group. Plasma levels of bicarbonate in the ADR group showed a biphasic response with an initial decrease and a later increase (Table 2). This group received significantly more insulin than the MIL or the CON group (Figure 2).

### Renal function and urinary excretion of alpha-1-microglobulin

The time courses of renal functional parameters and A1-MG_U _are given in Table 3, showing that A1-MG_U _was higher in the ADR than in the CON group 6 to 14 hours after ICU admission.

**Table 3:** Time course of renal functional variables and alpha-1-microglobuline excretion

		t0	t2	t6	t10	t14
UV (milliliters per minute)	ADR	3.3 ± 1.3^b^	2.7 ± 1.3	1.7 ± 0.7^a^	1.6 ± 0.7^a^	1.3 ± 0.5^a^
	MIL	3.2 ± 1.9^b^	3.5 ± 2.8	2.1 ± 0.8^a^	1.5 ± 0.4^a^	1.4 ± 0.6^a^
	CON	5.6 ± 2.6	4.8 ± 2.3	2.5 ± 0.9^a^	1.9 ± 0.6^a^	1.6 ± 0.6^a^

C_Crea _(milliliters per minute)	ADR	82.1 ± 30.5	76.0 ± 16.5	111.7 ± 59.5	120.6 ± 61.5	121.2 ± 62.2
	MIL	132.1 ± 55.6	149.7 ± 87.8	119.2 ± 36.5	119.3 ± 40.4	116.4 ± 58.6
	CON	135 ± 62.5	134.7 ± 51.8	150.6 ± 53.9	152.7 ± 37.0	136.3 ± 51.0

FE_Na _(percentage)	ADR	4.0 ± 2.2	2.8 ± 1.8	1.3 ± 1.3^a^	1.6 ± 1.3^a^	1.4 ± 0.7^a^
	MIL	2.2 ± 1.1	2.4 ± 2.6	1.5 ± 1.1	0.9 ± 0.8^a^	1.0 ± 0.7^a^
	CON	2.9 ± 2.4	2.5 ± 1.9	1.5 ± 0.6^a^	1.1 ± 0.5^a^	1.3 ± 0.6^a^

A1-MG_U _(milligrams per mole of creatinine)	ADR	225 ± 118	292 ± 133^bc^	200 ± 85^ab^	154 ± 63^ab^	135 ± 60^ab^
	MIL	206 ± 73	186 ± 75	156 ± 67^a^	117 ± 51^ab^	105 ± 46^a^
	CON	208 ± 64	170 ± 67^a^	110 ± 48^a^	71 ± 28	72 ± 50^a^

The time course of renal function parameters (urine flow [UV], creatinine clearance [C_Crea_], fractional excretion of sodium [FE_Na_], and the urinary excretion of alpha-1-microglobuline [A1-MG_U_]) in patients who presented with myocardial dysfunction (cardiac index of less than 2.2 liters/minute per square meter despite optimization of mean arterial pressure and filling pressures) upon intensive care unit admission and who were treated with adrenaline (ADR) or milrinone (MIL) and of control patients (CON) not needing inotropic after coronary artery bypass grafting with cardiopulmonary bypass. ^a^Significant difference (*p *< 0.05) in comparison with baseline (t0); ^b^significant difference (*p *< 0.05) in comparison with the control group; ^c^significant difference (*p *< 0.05) between the adrenaline and the milrinone group.

### Plasma cystatin-C levels

**Figure 3:**
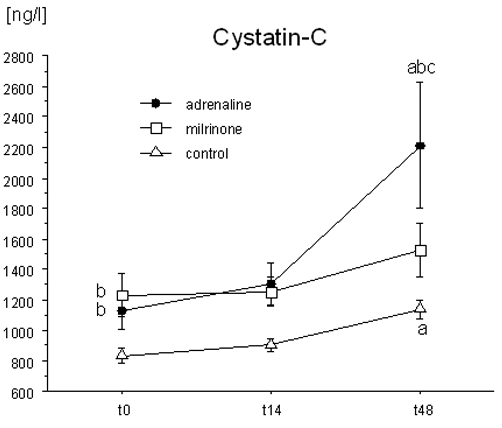
Plasma cystatin-C levels. The time course of plasma cystatin-C levels in patients with myocardial dysfunction after coronary artery bypass grafting surgery, treated with adrenaline (*n *= 7) or milrinone (*n *= 11), and in control patients (*n *= 20) not needing inotropic support. Data are given as mean ± standard error of the mean. ^a^Significant difference (*p *< 0.05) in comparison with baseline values (paired *t *test); ^b^significant difference (*p *< 0.05) in comparison with the control group (analysis of variance [ANOVA] with *post hoc *Fisher's predicted least-square difference [PLSD]); ^c^significant difference (*p *< 0.05) between the adrenaline and the milrinone group (ANOVA with *post hoc *Fisher's PLSD).

Cys-C plasma levels in the adrenaline- and the milrinone-treated patients were significantly higher than in the CON group at t0 and t14. At t48, Cys-C levels in the ADR and the CON group had increased significantly in comparison with baseline levels, whereas no significant increase in this parameter was observed in the MIL group. Therefore, plasma Cys-C levels at t48 were significantly higher in the ADR group than in the MIL and the CON group (Figure 3).

## Discussion

To the best of our knowledge, the present study is the first prospective and randomized trial comparing the effects of adrenaline and milrinone on metabolism and renal function in patients presenting with a low cardiac output state after ICU admission following cardiac surgery. Our data clearly show that the use of adrenaline – even in rather low doses – is associated with hyperlactatemia, hyperglycemia, delayed normalization of tubular proteinuria, and higher Cys-C levels in this situation.

### Metabolism

The relation between plasma lactate levels and mortality in patients undergoing cardiac surgery is not as clear as in the general ICU population. Nonetheless, increased lactate concentrations have been associated with a worse prognosis: In an observational study, Maillet and coworkers [13] have shown that patients presenting with hyperlactatemia upon ICU admission after cardiac surgery have increased mortality, that patients with late-onset hyperlactatemia have a higher rate of complications and a longer hospital stay than patients with normal lactate levels, and that adrenaline use and hyperglycemia are independent risk factors for postoperative hyperlactatemia.

An association between hyperglycemia, hyperlactatemia, and treatment with adrenaline has been described in a variety of settings, including healthy volunteers [14] and patients with sepsis and septic shock [15]. Additionally, Totaro and colleagues [16] have shown that 30% of patients receiving vasopressor doses of adrenaline after cardiac surgery developed lactic acidosis whereas plasma lactate in patients being resuscitated with noradrenaline remained unchanged. Furthermore, observational studies and case reports have shown an association between adrenaline use and hyperlactatemia [17,18]. However, this association has never been shown prospectively in patients needing inotropic support after cardiac surgery.

Adrenaline stimulates glycogenolysis and glycolysis, ultimately leading to an increase in ATP levels, as well as an activation of Na^+^/K^+^-ATPase. This leads to the consumption of ATP and the generation of ADP, stimulation of phosphofructokinase, further stimulation of glycolysis, and subsequent production of pyruvate that is converted into lactate by pyruvatdehydrogenase. Consequently, the increase in blood glucose and lactate levels may be regarded as a metabolic response to adrenaline stimulation [6].

In line with this, Levy and coworkers [19] have shown that muscle tissue is a major source of lactate in patients with sepsis during treatment with adrenaline. The authors showed that patients with septic shock had increased skeletal muscle lactate production and that these metabolic alterations could be blocked by the Na^+^/K^+^-ATPase blocker oubain, suggesting that the increased lactate levels were of a metabolic nature [19]. Consequently, the clinical relevance of increased lactate levels as a marker of ongoing cardiocirculatory dysfunction has been questioned by this group.

The present study does not allow us to determine whether this concept may also be applicable to the setting of postoperative LCOS. However, if the increase in lactate observed in this study after adrenaline treatment had been a purely metabolic response, it is to be expected that the relation between both parameters – the LPR – would not have changed. Interestingly, this was not the case. Immediately after the adrenaline treatment was started, the LPR was significantly higher than during milrinone treatment or control conditions. This suggests that a part of the lactate response after adrenaline may be not only an effect of increased metabolism but attributable to anaerobic metabolism in hypoperfused tissue despite seemingly normalized hemodynamics. This assumption is supported by a study performed immediately after CPB in patients with moderate hyperlactatemia [20] showing that intracellular ATP in muscle cells decreased whereas plasma and muscle lactate concentrations rose concomitantly. Unfortunately, we did not have the opportunity to measure regional perfusion in organs at risk for hypoperfusion after cardiac surgery and the source of excess lactate remains speculative.

Although it is still generally accepted that increased lactate levels in critically ill patients are associated with a worse prognosis, increased lactate levels may be, at least in part, a physiological response to severe stress. This can be derived from the experimental observation that lactate deprivation is associated with decreased cardiovascular performance and collapse in a rat model of endotoxin shock [21]. Comparably, it cannot be ruled out that high lactate levels may serve as a nutrient for other organs with a high need of ATP.

However, from the clinical point of view, the metabolic effects induced by adrenaline are in conflict with present strategies to achieve normoglycemia after cardiac surgery [22,23] and may also confound with lactate determinations during ongoing circulatory shock [24]. In contrast, the PDE-III inhibitor milrinone – despite being equieffective with respect to its inotropic effects – induced changes neither in plasma lactate or pyruvate nor in glucose levels in comparison with the control patients and in this regard may thus be the better choice for the treatment of myocardial dysfunction after CABG, even taking into account that patients in the MIL group needed slightly more noradrenaline than the CON group.

### Renal function

Renal dysfunction and renal failure are among the most important complications after cardiac surgery and are independently associated with a worse prognosis [25,26]. The mechanisms leading to postoperative renal dysfunction are multifactorial and incompletely understood. However, preoperative chronic kidney disease, duration of CPB and aortic crossclamp, volume depletion, preoperative reduced left ventricular ejection fraction, and prolonged low cardiac output states are among the most important risk factors for renal dysfunction in patients undergoing on-pump surgery [27]. Additionally, the inflammatory response induced by the CPB and the thoracotomy itself seem to play a relevant role [28,29]. Depending on the severity of the insult, the range of functional alterations extends from subtle changes in tubular function – detectable as an increase in urinary excretion of proteins normally reabsorbed by the tubular epithelium – to long-lasting changes in glomerular filtration rate, a decrease in sodium reabsorption capacity (that is, an increase in sodium excretion), and tubular necrosis.

A1-MG_U _is an accepted marker of renal tubular injury and has repeatedly been used for this purpose in patients undergoing cardiac surgery. Normally, A1-MG is glomerularily filtrated and reabsorbed to 95% at the proximal tubulus. A1-MG_U _has been shown to rise after release of the aortic crossclamp, but varying excretion patterns have been reported in the subsequent postoperative period [9,28,29]. Gormley and coworkers [28,29] observed a continuous decrease in the urinary A1-MG/creatinine ratio up to 24 hours with a subsequent further increase at 48 hours postoperatively. This contrasts with studies reported by Boldt and coworkers [9] showing a continuous rise in urinary A1-MG levels after surgery up to 48 hours. However, the latter data were not adjusted for creatinine concentration and thus may be influenced by the typical variations in UV observed after on-pump cardiac surgery (that is, a high UV immediately after surgery that normalizes after several hours).

The data derived from the present study suggest that the normalization of A1-MG/creatinine excretion in the ADR group, showing a steady decrease during the treatment period, was prolonged in comparison with the MIL and the CON group, suggesting a more severe form of proximal tubular injury during treatment with adrenaline. The higher rate of A1-MG_U _in the ADR group was not accompanied by changes in sodium excretion or C_Crea_. At first glance, this suggests that the observed alterations in tubular function did not become overt in terms of clinically detectable changes in renal concentration capability or glomerular filtration rate during the treatment period. However, sodium excretion as well as measured C_Crea _are subject to a high variance, at least in clinical situations, and thus may be too insensitive to detect more subtle renal dysfunction. To overcome this problem, we additionally determined plasma levels of Cys-C. Cys-C, a nonglycosylated peptide derived from nucleated cells, is glomerularily filtrated, reabsorbed, and almost completely catabolized by the proximal tubule. Consequently, the serum concentration of this peptide is directly related to glomerular filtration rate and more sensitive to acute changes in renal function than plasma creatinine [30].

At present, only few data are available about the natural course of Cys-C plasma levels in patients undergoing cardiac surgery [31,32]. The results of the present study offer some interesting new information:

Cys-C levels at t0 were significantly higher in both intervention groups, despite plasma creatinine levels, and C_Crea _did not even show a trend toward significant between-group differences at that time point. This suggests that, compared to conventional clinical parameters, Cys-C is indeed more sensitive to subtle changes in renal function as they may occur during a low output state.

Plasma Cys-C levels in the MIL group did not change during the treatment and the observation period. At the end of the observation period, Cys-C levels in the ADR group had almost doubled in comparison with t0 and were significantly higher than in the MIL and the CON group. This observation is highly suggestive that the use of adrenaline, in contrast to milrinone, is associated with renal dysfunction.

### Hemodynamics

We observed only minor differences in the course of cardiac output and SvO_2 _during the treatment period in the study groups, suggesting that we were indeed successful in establishing comparable hemodynamics and that the observed metabolic and renal changes cannot be explained by different circulatory states. However, the higher CVP at baseline and the later increase in HR in the MIL group – despite the fact that fluid balance was comparable to the CON and the ADR group – are suggestive that myocardial contractility was more depressed in the patients randomly assigned to MIL than in those treated with ADR. This, however, is speculative.

### Limitations

The major limitations of the present study are the small sample size and the uneven distribution of patients in the intervention groups. However, despite this, the between-group differences were so pronounced that it is rather unlikely that a larger sample size would have changed the results. The small sample size and the uneven group sizes are the consequence of our difficulties to enroll the intended number of 20 patients per group within the study period of 18 months. This, however, is the direct result of intensified perioperative monitoring since more than 30% of patients needed intraoperative inotropic support, many of these already before CPB. Consequently, only a few patients presented with myocardial dysfunction in the ICU.

## Conclusion

The data derived from this pilot study suggest that the use of adrenaline in patients needing inotropic support after cardiac surgery – in contrast to treatment with the PDE-III inhibitor milrinone – is associated with unwarranted metabolic and detrimental renal effects. In line with observational data showing an increased mortality in critically ill patients treated with adrenaline, this questions the appropriateness of the current practice in German heart centers of using adrenaline as a first-line agent in patients presenting with myocardial dysfunction after CABG surgery.

## Key messages

• Treatment with adrenaline in patients with myocardial dysfunction after coronary artery bypass surgery is associated with higher plasma lactate and glucose levels and increased insulin needs in comparison with the phosphodiesterase III inhibitor milrinone.

• The use of adrenaline – in contrast to milrinone – leads to delayed normalization of the urinary excretion of alpha-1-microglobulin and an increase in plasma levels of cystatin-C, suggesting that adrenaline aggravates perioperative tubular injury and leads to a decrease in glomerular filtration rate that may not be readily detected by the course of the plasma creatinine concentration.

• The results of this prospective, randomized pilot study thus question the appropriateness of using adrenaline as a first-line agent in patients with myocardial dysfunction after on-pump coronary artery surgery.

## Abbreviations

A1-MG = alpha-1-microglobuline; A1-MG_U _= urinary excretion of alpha-1-microglobuline; ADR = adrenaline; CABG = coronary artery bypass grafting; C_Crea _= creatinine clearance; CI = cardiac index; CON = control; CPB = cardiopulmonary bypass; CVP = central venous pressure; Cys-C = cystatin-C; HR = heart rate; ICU = intensive care unit; LCOS = low cardiac output syndrome; LPR = lactate/pyruvate ratio; MAP = mean arterial blood pressure; MIL = milrinone; PDE-III = phosphodiesterase III; SvO_2 _= mixed venous oxygen saturation; UV = urine flow.

## Competing interests

The authors declare that they have no competing interests.

## Authors' contributions

MH, HH, and JS participated in the design of the study, performed the statistical analyses, and were responsible for drafting the manuscript. MW and JG performed the data acquisition and calculations and were involved in drafting the manuscript. SK, MB, and LB participated in coordinating the study and in the interpretation of data and were involved in drafting the manuscript. JP was involved in the interpretation of the data and revised the manuscript for important intellectual content. All authors read and approved the final manuscript.
